# Differences in life-history and ecological traits between co-occurring *Panulirus* spiny lobsters (Decapoda, Palinuridae)

**DOI:** 10.3897/zookeys.457.6669

**Published:** 2014-11-25

**Authors:** Patricia Briones-Fourzán

**Affiliations:** 1 Universidad Nacional Autónoma de México, Instituto de Ciencias del Mar y Limnología, Unidad Académica de Sistemas Arrecifales. Prol. Av. Niños Héroes s/n, Puerto Morelos, Quintana Roo, México

**Keywords:** Crustacea, Decapoda, Achelata, Palinuridae, coexistence, resource use, predation, Eastern Central Pacific, Caribbean region

## Abstract

Coexistence of closely related species may be promoted by niche differentiation or result from interspecific trade-offs in life history and ecological traits that influence relative fitness differences and contribute to competitive inequalities. Although insufficient to prove coexistence, trait comparisons provide a first step to identify functional differences between co-occurring congeneric species in relation to mechanisms of coexistence. Here, a comparative review on life history and ecological traits is presented for two pairs of co-occurring species of spiny lobsters in the genus *Panulirus*: *Panulirus gracilis* and *Panulirus inflatus* from the Eastern Central Pacific region, and *Panulirus argus* and *Panulirus guttatus* from the Caribbean region. *Panulirus gracilis* and *Panulirus inflatus* have similar larval, postlarval, and adult sizes and a similar diet, but differ in degree of habitat specialization, fecundity, and growth rate. However, little is known on behavioral traits of these two species that may influence their competitive abilities and susceptibility to predators. The more abundant information on *Panulirus argus* and *Panulirus guttatus* shows that these two species differ more broadly in degree of habitat specialization, larval, postlarval and adult sizes, diet, fecundity, growth rate, degree of sociality, defense mechanisms, susceptibility to predators, and chemical ecology, suggesting a greater degree of niche differentiation between *Panulirus argus* and *Panulirus guttatus* than between *Panulirus gracilis* and *Panulirus inflatus*. Whether the substantial niche differentiation and apparent interspecific trade-offs between *Panulirus argus* and *Panulirus guttatus* relative to *Panulirus gracilis* and *Panulirus inflatus* reflect an earlier divergence of the former pair of species in the evolution of the genus constitutes an intriguing hypothesis. However, whether or not post-divergence evolution of each species pair occurred in sympatry remains uncertain.

## Introduction

Spiny lobsters (Decapoda: Achelata: Palinuridae) are large (body length up to 60 cm), long-lived (> 10 years) crustaceans that occur in a wide range of habitats and depths, and constitute some of the most important fishing resources in all parts of the world (Phillips 2006). Spiny lobsters exhibit complex behaviors and are an important component of community structure and function because they consume a vast array of small benthic organisms and are prey to numerous species of higher predators (Lipcius and Eggleston 2000, Phillips et al. 2013). In addition, spiny lobsters are sturdy and easy to keep in controlled laboratory conditions, making them useful subjects for many types of biological, physiological, and behavioral studies (Cobb 2006).

The family Palinuridae comprises 54 extant species/subspecies arranged in 12 genera (Chan 2010), all of which have a specialized, flat-bodied larva called phyllosoma with multiple stages and a long (4–22 months) planktonic life, as well as a swimming, non-feeding postlarva called puerulus. The most diverse genus is *Panulirus*, with 24 species/subspecies, followed by *Jasus* and *Palinurus*, with six species each. These three genera contain the great majority of commercially important species. However, *Jasus* species are distributed exclusively in cold waters of the southern hemisphere (Jeffs et al. 2013), whereas *Palinurus* species are restricted to south east Africa and the north-eastern Atlantic, and generally occur at depths greater than 100 m (Groeneveld et al. 2013). In contrast, *Panulirus* species occur in shallow tropical and subtropical waters (< 100 m in depth) of both hemispheres, where the diversity of habitats may have promoted a greater radiation of this genus (George and Main 1967, George 2006). Therefore, the occurrence of two or more *Panulirus* species living in sympatry is common in different regions throughout the world (Briones-Fourzán and Lozano-Álvarez 2013).

The co-occurrence of congeneric species at local scales is common in many marine systems (e.g. Azovsky 1996, Sfenthourakis et al. 2005, González et al. 2011), but co-occurrence does not necessarily imply coexistence. The key criterion for coexistence is the “invasibility” criterion, which requires that each species must be able to increase from low density (i.e. persist) when the others are at their typical abundances (Chesson 2000). Conditions that are necessary but not sufficient for invasibility include negative density dependence and trade-offs in performance that influence population regulation (Siepielski and McPeek 2010). Trade-offs imply that advantages that one species may have over others are offset by compensating disadvantages (Chesson 2000, Kneitel and Chase 2004). For example, coexistence of species may be promoted by trade-offs between competitive ability and dispersal ability, between abilities to compete for alternative resources, between competitive ability and disturbance tolerance, and between competitive ability and susceptibility to predation or disease (Bohannan et al. 2002, Amarasekare 2003, 2008, Kneitel and Perrault 2006). These trade-offs may involve niche differentiation between species (McPeek 1996) or may result from interspecific trade-offs in life history and ecological traits that influence relative fitness differences and contribute to competitive inequalities (e.g. body size, fecundity, longevity, dispersal) (Tilman 1994, Chesson 2000, Amarasekare 2003, Amarasekare et al. 2004).

Because of the wealth of data needed, it is difficult to prove whether co-occurring species truly coexist, particularly for long-lived species wherein the relevant data should have to span multiple generations of each species (Siepielski and McPeek 2010, HilleRisLambers et al. 2012, Narwani et al. 2013). On the other hand, many studies related to coexistence have addressed species belonging to different genera, underscoring the need for more studies focusing on trying to understand the degree to which congeneric species that are within the same trophic level coexist, in particular at local scales (Siepielski and McPeek 2010). As species within genera are often predicted to be more similar to each other than between genera, trait-based approaches may provide a first step to identify functional differences between co-occurring congeneric species in relation to mechanisms of coexistence (Tilman 1994). Therefore, the aim of the present review is to compare life history and ecological traits between some coexisting *Panulirus* species as a first step to suggesting potential trade-offs that may promote their coexistence.

**Figure 1.**
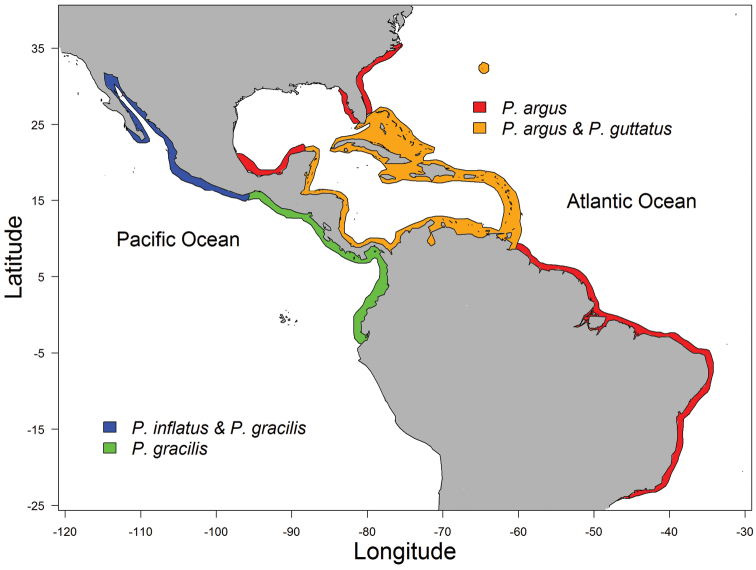
Geographic distribution of the two pairs of sympatric *Panulirus* species addressed in the text.

There are numerous studies addressing biological and/or ecological traits of spiny lobsters but few studies comparing traits between co-occurring species. For example, the co-occurrence of multiple *Panulirus* species in tropical waters of the Indo-West Pacific has been related to a differential use of habitats of adult lobsters across environmental gradients such as depth, turbidity, coral cover, and wave action (de Bruin 1969, Berry 1971, George 1974, Pitcher 1993, Coutures and Chauvet 2003), but there is little information on the life-history traits of these particular species. Therefore, emphasis is made in this review on two pairs of co-occurring congeneric species for which relatively more information is available, one from the Eastern Central Pacific region (*Panulirus gracilis* Streets, 1871 and *Panulirus inflatus* (Bouvier, 1895)) and the other from the Caribbean region (*Panulirus argus* (Latreille, 1804) and *Panulirus guttatus* (Latreille, 1804)) (Fig. 1). Throughout the text, measurements are given as mean ± 95% confidence interval unless otherwise stated.

## Eastern Central Pacific: *Panulirus gracilis* and *Panulirus inflatus*

*Panulirus gracilis* occurs along the continental coast and islands from Peru to Mexico, and co-occurs with *Panulirus inflatus* along most of the Pacific coast of Mexico (Holthuis 1991) (Fig. 1). These two species are so similar that they were considered as synonyms until Holthuis and Villalobos (1961) established that they constituted separate species. During 1976–1980, the biology, ecology and fisheries of both species were concurrently studied in Zihuatanejo, Mexico, by researchers from the National Autonomous University of Mexico. Comparative analyses of the original data from these and other studies provide insight into some differences in life history and ecological traits between these two species.

### Larval and postlarval traits

Both *Panulirus inflatus* (see Johnson and Knight 1966) and *Panulirus gracilis* (see Báez 1983) have eleven phyllosoma stages which, according to Johnson (1971), are almost identical in almost every respect, except for the presence of a subexopodal spine on the fourth pereopod in stages five through eleven of *Panulirus gracilis* and the absence of this spine in all stages of *Panulirus inflatus*. However, molecular analyses have shown that this and other morphological criteria are insufficient to distinguish between phyllosomata of the two species (García-Rodríguez et al. 2008). The length of the larval phase has not been determined for either species.

Johnson (1971) described the puerulus of “*Panulirus inflatus*-*gracilis*” from plankton samples collected over an area where the two species co-occur, whereas Báez (1983) described the puerulus of *Panulirus gracilis* from samples collected in an area where only this species occurs. Both pueruli are similar in size (7.0–8.9 mm carapace length, CL) and have long, spatulated antennae, which are 2.7 times the length of the body in *Panulirus gracilis* (see Báez 1983) and about 2 times the length of the body in “*Panulirus inflatus*-*gracilis*” (see Johnson 1971). Based on these and other minor differences, Báez (1983) suggested that the puerulus of “*Panulirus inflatus*-*gracilis*” described by Johnson (1971) belonged to *Panulirus inflatus*.

### Life history strategy and benthic distribution

*Panulirus gracilis* occupies different types of benthic habitats, from rocky bottoms with clear water to gravel-sand bottoms near river discharges where water can be considerably turbid, whereas *Panulirus inflatus* occurs exclusively in rocky habitats with clear waters (Briones et al. 1981, Pérez-González et al. 1992, Pérez-González 2011). Although these findings suggest that *Panulirus inflatus* might be considered a habitat specialist and *Panulirus gracilis* a habitat generalist, an important criterion to consider a benthic species as habitat specialist is that its postlarvae settle into the same type of habitat where the adults live, and as habitat generalist that the postlarvae are able to settle in various types of habitats (Adams et al. 2006, Adams and Ebersole 2009); however, the natural settlement habitats have not been determined for either *Panulirus inflatus* or *Panulirus gracilis*. However, recently settled pueruli and early benthic juveniles of both species (6-24 mm carapace length, CL) were consistently found co-occurring among the profuse biota fouling the pylons of concrete piers in Zihuatanejo, but those of *Panulirus inflatus* persist longer in this particular habitat, which resembles the rocky habitats occupied by *Panulirus inflatus* adults (Briones-Fourzán and Lozano-Álvarez 2013). Also, Gracia and Lozano (1980) found numerous pueruli in the stomachs of benthic catfish *Occidentarius platypogon* (Günther, 1864) (previously known as *Netuma platypogon*), which they assigned to “*Panulirus inflatus*-*gracilis*” following Johnson (1971). However, it is possible that those pueruli belonged to *Panulirus gracilis* which, unlike *Panulirus inflatus*, dwells in the same benthic habitats as *Occidentarius platypogon* (gravel-sand and muddy bottoms).

### Body size, growth rate, and fecundity

**Figure 2.**
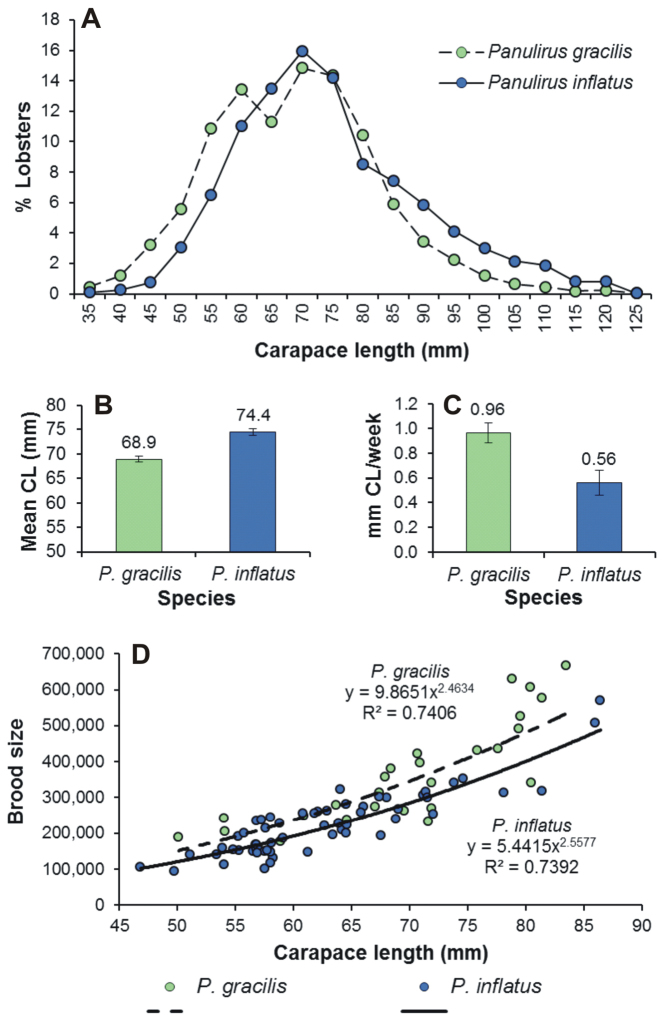
Differences in some life-history traits between *Panulirus gracilis* and *Panulirus inflatus* from Zihuatanejo, Mexico. **A** carapace length (CL) distribution (n *Panulirus gracilis*: 2162, n *Panulirus inflatus*: 1873) **B** mean size **C** growth rate of males (mm CL week^–1^, n *Panulirus gracilis*: 148, n *Panulirus inflatus*: 34) **D** brood size (number of eggs per clutch) versus CL relationship. Error bars denote 95% confidence intervals. (Data from **A, B** Briones-Fourzán and Lozano-Álvarez 1992, **C** Briones-Fourzán and Lozano-Álvarez 2003, **D** Gracia 1985, Fernández-Lomelín 1992).

Adults of *Panulirus inflatus* and *Panulirus gracilis* reach a similar body size (Fig. 2A). In Zihuatanejo, *Panulirus inflatus* has a slightly larger mean size (CL) than *Panulirus gracilis* (Fig. 2B), but mark-recapture data showed that *Panulirus gracilis* grows almost twice as fast as *Panulirus inflatus* (e.g. growth rate for males, *Panulirus gracilis*: 0.96 ± 0.08 mm CL week^–1^, *Panulirus inflatus*: 0.56 ± 0.10 mm CL week^–1^, Briones-Fourzán and Lozano-Álvarez 2003) (Fig. 2C). In this same location, the size of the smallest ovigerous females ever recorded and the size at which 50% of females are ovigerous (CL_50_) were slightly larger for *Panulirus gracilis* (47.5 mm CL and 80.0 mm CL, respectively) than for *Panulirus inflatus* (45.6 mm CL and 77.5 mm CL, respectively) (Weinborn 1977, Gracia 1985, Briones-Fourzán and Lozano-Álvarez 1992). Both species have an extended yearly reproductive period during which individual females can produce up to four clutches (and possible more), with embryo development taking approximately three to four weeks before hatching (Briones-Fourzán and Lozano-Álvarez 1992, Torres-Zepeda et al. 2008). Using original data on clutch size (number of eggs) versus CL concurrently taken for *Panulirus inflatus* (see Gracia 1985) and *Panulirus gracilis* (see Fernández-Lomelín 1992) from Zihuatanejo (Fig. 2D), an analysis of covariance (ANCOVA) showed that, after controlling for the significant effect of CL (*F*_1,82_ = 231.71, *p* < 0.0001), size-specific fecundity is significantly greater in *Panulirus gracilis* than in *Panulirus inflatus* (*F*_1,82_ = 16.24, *p* < 0.001) (Fig. 2D). Large broods are achieved partly through selection for smaller egg size. Pollock (1997) showed that, for spiny lobsters and other crustaceans, the number of eggs per gram of body weight provides an inverse index of egg size (i.e., the larger the index the smaller the egg). The use of this index shows that the eggs of *Panulirus gracilis* (1047 ± 87 eggs g^–1^ body weight) are indeed significantly smaller than those of *Panulirus inflatus* (911 ± 54 eggs g^–1^ body weight) (Student’s *t*-test, *t*_85_ = 2.685, *p* = 0.009).

### Use of habitat resources

**Figure 3.**
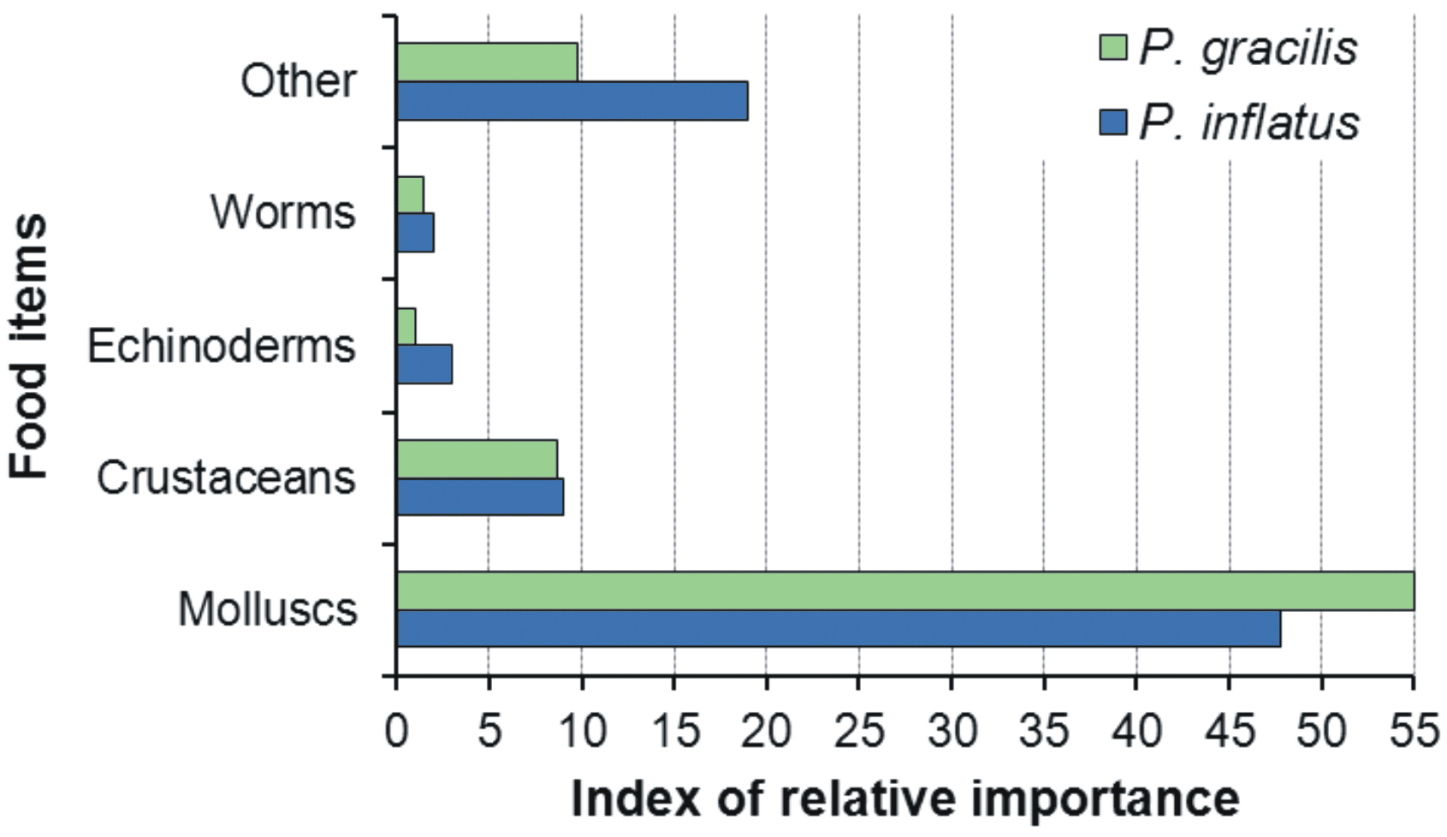
Diet of *Panulirus gracilis* and *Panulirus inflatus* from Zihuatanejo, Mexico. For each food item the index of relative importance (IRI) is estimated as IRI = (% frequency × % weight)/100. (Data from Lozano-Álvarez and Aramoni-Serrano 1996).

**Figure 4.**
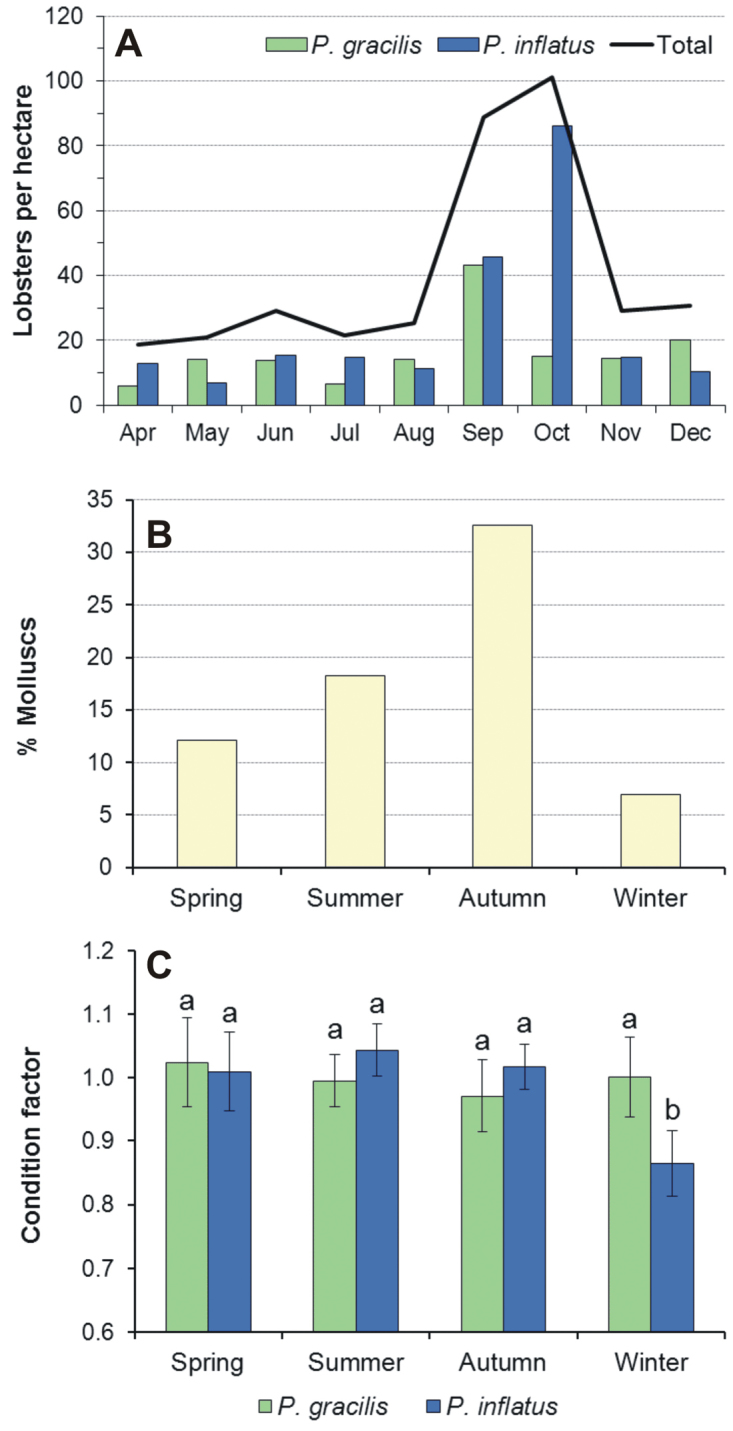
Potential ecological interactions between *Panulirus gracilis* and *Panulirus inflatus* in a rocky site (“Site A”) in Zihuatanejo, Mexico. **A** lobster density (number of individuals ha^–1^) **B** relative abundance of molluscs (percentage of molluscs in benthic samples) **C** condition factor of lobsters. Error bars denote 95% CI. (Data from **A** Lozano et al. 1982, **B** Aramoni-Serrano 1982, **C** Lozano-Álvarez and Aramoni-Serrano 1996).

Stomach content analyses showed that *Panulirus gracilis* and *Panulirus inflatus* consume various types of invertebrate prey but that both species exhibit a marked preference for molluscs (Lozano-Álvarez and Aramoni-Serrano 1996) (Fig. 3). In Zihuatanejo, a capture-recapture experiment was conducted during 1979–80 to estimate monthly lobster densities on a 36-ha rocky site (“site A”) where the two species co-occurred (Lozano et al. 1982). At the same time, the seasonal composition of the benthic community at site A and other sites, as well as the seasonal changes in condition factor of the two lobster species were studied (Aramoni-Serrano 1982, Lozano-Álvarez and Aramoni-Serrano 1996). The total density of lobsters on site A showed a marked increase in September-October relative to the other months (Fig. 4A). For each separate species, the density showed values ≤ 15 ind. ha^–1^ between April and August, but then more than doubled in September. In October, the density of *Panulirus inflatus* doubled again while that of *Panulirus gracilis* decreased to previous levels. By November, the density of *Panulirus inflatus* also decreased to previous levels (Fig. 4A).

Interestingly, site A (but not other sites) exhibited a peak in relative abundance of molluscs in the autumn (Fig. 4B), suggesting that this particular site became a food-rich habitat patch for lobsters during this season (Aramoni-Serrano 1982) and potentially explaining the substantial increase in local density of both *Panulirus inflatus* and *Panulirus gracilis* in September, which possibly reached the carrying capacity of the site. In October, the increase in density of *Panulirus inflatus* and decrease in density of *Panulirus gracilis* was followed by the recapture of several *Panulirus gracilis* lobsters during October to December on a gravel-sand site 3 km away from site A (Lozano et al. 1982), suggesting that *Panulirus inflatus* was able to displace *Panulirus gracilis* from the food-rich patch. However, the high density of *Panulirus inflatus* during the autumn possibly resulted in intraspecific competition for food resources, further exacerbated by the local decrease in abundance of molluscs during the winter (Lozano-Álvarez and Aramoni-Serrano 1996), as indicated by the significantly lower condition factor of *Panulirus inflatus* during the winter relative to other seasons and to *Panulirus gracilis* (Fig. 4C). In conjunction, these findings suggest that *Panulirus inflatus* is the superior competitor in the rocky habitats to which this species is restricted.

## Greater Caribbean region: *Panulirus argus* and *Panulirus guttatus*

*Panulirus argus* and *Panulirus guttatus* co-occur throughout the Greater Caribbean region (see Fig. 1). Some of the following sections are based on studies on these lobsters conducted by researchers from the National Autonomous University of Mexico in the Caribbean coast of Mexico, where life history traits and ecological aspects of these species have been concurrently studied for over 20 years.

### Larval and postlarval traits

Lewis (1951) described eleven phyllosoma stages for *Panulirus argus* from plankton samples and estimated the larval duration in about six months, but Goldstein et al. (2008), who obtained the complete larval phase in the laboratory, identified only ten distinct stages with the entire larval duration in these controlled conditions varying between 4.6 and 6.6 months. The early phyllosoma stages of *Panulirus guttatus* have not been described and the larval duration of this species has not been determined. According to Chitty (1973), first-stage phyllosomata of *Panulirus argus* and *Panulirus guttatus* are virtually indistinguishable based on morphology and size. However, stages VI to X of *Panulirus guttatus* are substantially larger than the corresponding stages of *Panulirus argus* (Baisre and Alfonso 1994).

The puerulus of *Panulirus argus* is relatively small (6.1 mm CL on average) and has tapered antennae about 1.5 times the length of the body (Lewis et al. 1952, Goldstein et al. 2008). In contrast, the puerulus of *Panulirus guttatus* is quite large (10 mm CL) and has long, spatulated antennae about 2.5 times the length of the body (Briones-Fourzán and McWilliam 1997). However, upon molting into the first juvenile stage and as individuals continue to grow, the antennae of *Panulirus guttatus* become progressively shorter and thinner than those of *Panulirus argus* (Briones-Fourzán et al. 2006).

### Life history strategy and benthic distribution

It is well known that *Panulirus argus* is an ontogenetic shifter, i.e. a species wherein the postlarvae settle into habitats distinct from those of the adults and further undergo notable ontogenetic habitat shifts toward the adult habitat (Adams and Ebersole 2009). The pueruli of *Panulirus argus* settle in vegetated habitats of shallow reef lagoons and bays (seagrass meadows, macroalgal beds, coastal mangroves), where the early benthic juveniles remain for a few months, taking shelter among the vegetation. Eventually, juveniles outgrow the protection afforded by the vegetation and seek shelter in any crevice-type structure in or adjacent to the settlement habitats before gradually moving to the coral reef habitats where the subadults and adults dwell (Butler et al. 2006). Mating and brooding of *Panulirus argus* occur in the reef habitat, but after embryo development – which takes from three to four weeks – is completed, the females move to deeper areas to release the phyllosoma larvae (Bertelsen 2013), which develop in oceanic waters.

Upon changing habitats, ontogenetic shifters also tend to undergo changes in behavior (Adams et al. 2006). Indeed, after their first benthic habitat shift, *Panulirus argus* lobsters change from being asocial to being highly gregarious, with multiple individuals commonly sharing individual crevice shelters (Childress and Herrnkind 1996). In addition, *Panulirus argus* has a highly mobile lifestyle, with movement ranges increasing with lobster size. In some locations, these movements include organized mass migrations over tens to hundreds of kilometers (Herrnkind 1969).

By contrast, *Panulirus guttatus* is a habitat specialist, as the pueruli of this species settle directly into the coral reef habitat where the juveniles and adults also dwell (Briones-Fourzán and McWilliam 1997). Individuals of *Panulirus guttatus* are highly sedentary, with a home range for adults of approximately 100 m in radius (Lozano-Álvarez et al. 2002). Therefore, growth, mating, brooding, and egg hatching all take place in the coral reef habitat (Briones-Fourzán and Contreras-Ortiz 1999, Negrete-Soto et al. 2002). Individuals of *Panulirus guttatus* use reef crevices as shelters, and although small groups can share crevices, many individuals dwell solitarily, reflecting a much lower degree of gregariousness than that exhibited by *Panulirus argus* (Briones-Fourzán 1995, Sharp et al. 1997, Briones-Fourzán and Lozano-Álvarez 2008).

### Body size, growth rate, and fecundity

**Figure 5.**
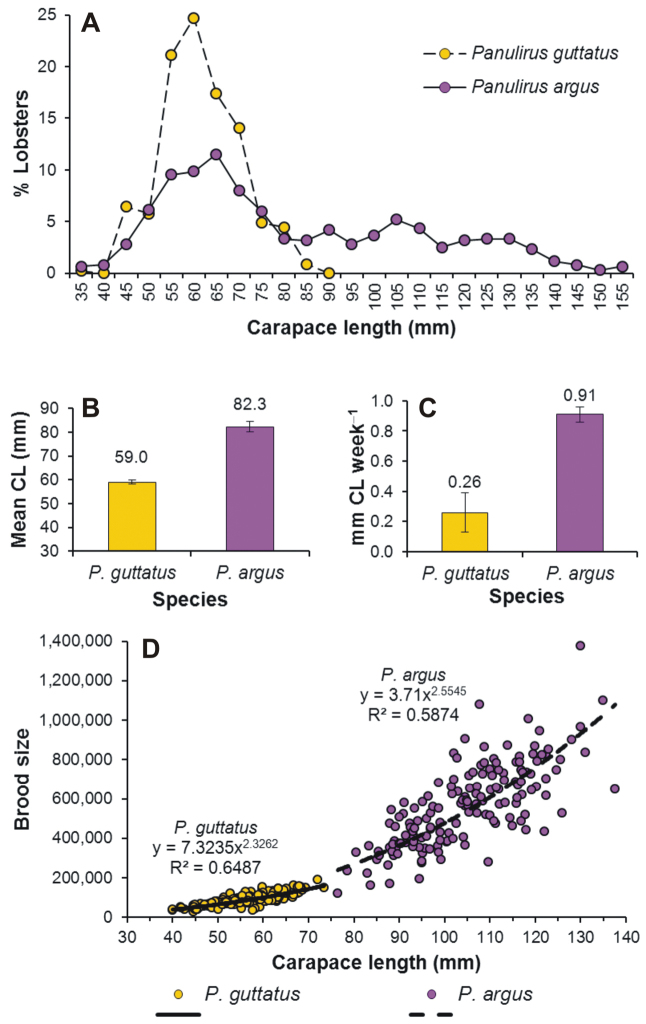
Differences in some life-history traits between *Panulirus argus* and *Panulirus guttatus* from Puerto Morelos, Mexico. **A** carapace length (CL) distribution (n *Panulirus argus*: 717, n *Panulirus guttatus*: 450) **B** mean size **C** growth rate of males (mm CL week^–1^, n *Panulirus argus*: 148, n *Panulirus guttatus*: 57) **D** brood size (number of eggs per clutch) versus CL relationship. Error bars denote 95% confidence intervals. (Data from **A, B** Lozano-Álvarez et al. 2007, Briones-Fourzán and Lozano-Álvarez 2013, **C** Negrete-Soto et al. 2002, **D** Fonseca-Larios and Briones-Fourzán 1998, Briones-Fourzán and Contreras-Ortiz 1999).

Adults of *Panulirus argus* and *Panulirus guttatus* have a very different body size (Fig. 5A). For example, in the Puerto Morelos coral reef, *Panulirus argus* has a much larger mean size (82.3 ± 2.24 mm CL) than *Panulirus guttatus* (59.0 ± 0.83 mm CL) (Fig. 5B) and the former species also grows much faster than the latter (weekly growth rate for males, *Panulirus argus*: 0.91 ± 0.6 mm CL week^–1^, *Panulirus guttatus*: 0.26 ± 0.13 mm CL week^–1^) (Lozano-Álvarez et al. 1991, Negrete-Soto et al. 2002) (Fig. 5C). In the same location, the largest ovigerous female of *Panulirus guttatus* ever recorded (73.5 mm CL) was smaller than the smallest ovigerous female of *Panulirus argus* ever recorded (75.0 mm CL). In both species, large females can produce up to four broods per year (Cruz and de León 1991, Briones-Fourzán and Contreras-Ortiz 1999), but the CL_50_ of ovigerous females is 95.5 mm CL for *Panulirus argus* and 59.0 mm CL for *Panulirus guttatus* (Briones-Fourzán 1995). Due to the large interspecific difference in size, size-specific fecundity is far larger in *Panulirus argus* than in *Panulirus guttatus* (Fonseca-Larios and Briones-Fourzán 1998, Briones-Fourzán and Contreras-Ortiz 1999) (Fig. 5D), more so when the size of the eggs is taken into account. As Pollock (1997) had previously noted, the number of eggs per gram of body weight is significantly larger (indicating smaller eggs) in *Panulirus argus* (689 ± 27) than in *Panulirus guttatus* (519 ± 15) (*t*_322_ = 10.925, p < 0.0001).

### Use of reef resources

**Figure 6.**
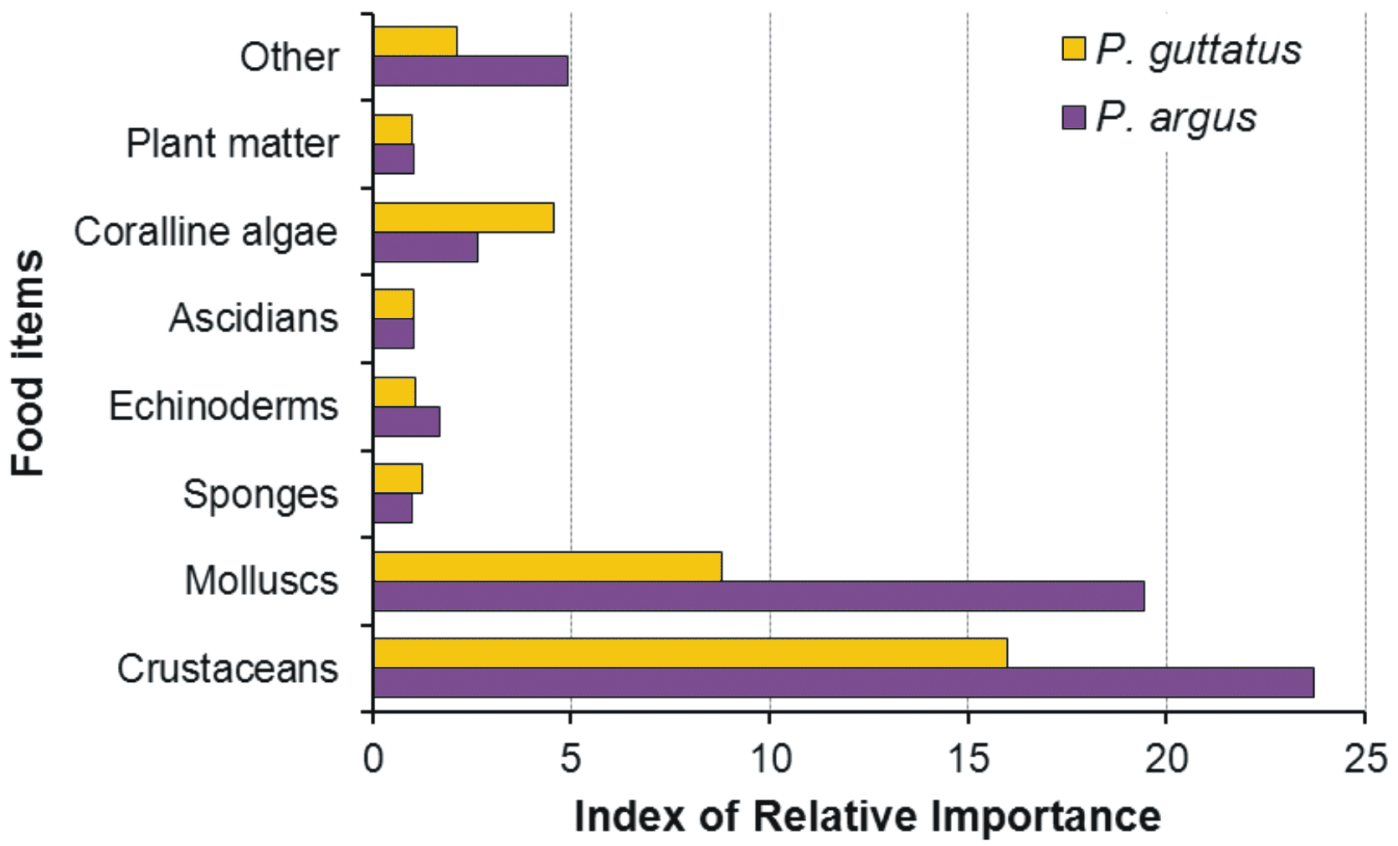
Diet of *Panulirus argus* and *Panulirus guttatus* from Puerto Morelos, Mexico. For each food item the index of relative importance (IRI) is estimated as IRI = (% frequency × % weight)/100. (Data from Colinas-Sánchez and Briones-Fourzán 1990).

The benthic distribution of *Panulirus argus* and *Panulirus guttatus* overlaps in the coral reef habitat. In Puerto Morelos, *Panulirus guttatus* outnumbers *Panulirus argus* by 5 to 1 across the entire reef habitat, but the relative density of each species varies with reef zone. Thus, the ratio of *Panulirus guttatus* to *Panulirus argus* is, on average, 2:1 in the back reef (the protected reef zone facing the mainland), but 16:1 in the fore reef (the exposed reef zone facing the open waters) (Lozano-Álvarez et al. 2007). A numerical dominance of *Panulirus guttatus* over *Panulirus argus* on fore reefs has also been reported in Florida (Sharp et al. 1997) and Belize (Acosta and Robertson 2003). However, there is no evidence that *Panulirus guttatus* can displace *Panulirus argus* via interference competition because individuals of these congeneric species do not act aggressively toward each other even when in close proximity (Lozano-Álvarez and Briones-Fourzán 2001). Rather, there is evidence that *Panulirus guttatus* and *Panulirus argus* make a differential use of reef resources (Lozano-Álvarez et al. 2007). For example, although lobsters of both species feed on a wide variety of organisms with a marked preference for crustaceans and molluscs (Colinas-Sánchez and Briones-Fourzán 1990) (Fig. 6), interspecific competition for food resources is unlikely, as individuals of *Panulirus guttatus* forage on the reef itself (Wynne and Côté 2007) whereas reef-dwelling individuals of *Panulirus argus* forage on seagrass and soft-bottom areas adjacent to the coral reefs (Cox et al. 1997, Briones-Fourzán et al. 2003).

Also, *Panulirus argus* lobsters tend to occupy crevices (‘dens’) along the lower and middle portions of the reef and *Panulirus guttatus* lobsters over the middle and upper portions of the reef (Lozano-Álvarez et al. 2007). Occupation of individual dens by multiple conspecifics is more common for *Panulirus argus*, whereas occupation of individual dens by solitary individuals is more common for *Panulirus guttatus*. Moreover, in dens harboring either species separately or both species together, *Panulirus argus* lobsters typically occupy the floor and the entrances of the dens, while *Panulirus guttatus* lobster typically occupy the deep recesses of the dens, clinging to the ceiling or walls (Sharp et al. 1997, Lozano-Álvarez et al. 2007, Briones-Fourzán and Lozano-Álvarez 2008). The differential pattern of occupation of the den space by these congeners appears to minimize competition for shelter and to be related with a differential susceptibility to predators.

### Susceptibility to predators

An important ecological trade-off that favors local coexistence of similar species within the same trophic level is a differential susceptibility to predators (Amarasekare 2008). This has been explored for *Panulirus guttatus* and *Panulirus argus*, which are potential prey to the same predators in the coral reef habitat (e.g. Randall 1967). In field studies, a negative relationship between the abundances of predators and prey would suggest that the abundance of predators controls the abundance of prey (e.g. Sih 1984, Hixon and Beets 1993, Eggleston et al. 1997). In the Puerto Morelos coral reef, Lozano-Álvarez et al. (2007) examined the relationship between the densities of predators and lobsters of each species by reef zone. A negative relationship emerged only for *Panulirus guttatus* at the fore-reef zone, where this species was numerically dominant.

In the laboratory, individuals of *Panulirus guttatus* and *Panulirus argus* differed significantly in performance of several defense mechanisms expressed by spiny lobsters, indicating a differential defense strategy for each species (Briones-Fourzán et al. 2006). In particular, *Panulirus argus* relies heavily on the formidable defense of its long, strong spiny antennae, especially when multiple individuals join in cooperative defense, whereas *Panulirus guttatus*, which has much thinner and weaker antennae, does not express communal defensive behavior at all. *Panulirus guttatus* lobsters are also more cryptic and only emerge from their shelters to forage for short periods during the night. Individuals of *Panulirus argus* have to traverse open areas to forage and tend to remain stationary to minimize detection when a predator approaches. If a predator attacks, a *Panulirus argus* lobster can confront the predator, turning deftly to face it at all times, lashing and raking at the predator with its antennae. By contrast, if an individual of *Panulirus guttatus* detects an approaching predator, it retreats backwards into the nearest available reef crevice, and if attacked, it can swim backwards in a slow but protracted bout that will effectively remove it from the visual field of the predator. In essence, *Panulirus guttatus* exhibits a shy behavioral type and a higher susceptibility to predators, whereas *Panulirus argus* exhibits a bold behavioral type and a lower susceptibility to predators (Briones-Fourzán et al. 2006).

### Chemical ecology

The behavior of spiny lobsters is largely mediated by chemical communication (Aggio and Derby 2011). Because shelter is a limiting resource for these lobsters, individuals that are seeking shelter tend to be attracted to chemical scents released by sheltered conspecifics (“attraction cues”). On the other hand, avoiding scents from a lethally injured or freshly killed conspecific (“alarm odors”), which may signal the proximity of a predator, is a particularly effective antipredator strategy for gregarious species (Dicke and Grostal 2001). However, the degree of gregariousness varies widely among spiny lobsters (Childress 2007) and is particularly different between *Panulirus argus* and *Panulirus guttatus* (see Briones-Fourzán and Lozano-Álvarez 2008).

Briones-Fourzán et al. (2008) compared how individuals of *Panulirus argus* and *Panulirus guttatus* responded to attraction cues and alarm odors from either conspecifics or congeners. As expected, individuals of both species were significantly attracted to shelters emanating conspecific attraction cues but responded neutrally to shelters emanating congeneric attraction cues. However, individuals of *Panulirus guttatus* responded neutrally to shelters emanating either conspecific or congeneric alarm odors, whereas individuals of *Panulirus argus* significantly avoided shelters emanating either conspecific or congeneric alarm odors. The differential responses to alarm odors between species suggest that the cryptic defensive behavior of *Panulirus guttatus* appears to be sufficiently adaptive to offset the need to avoid dens with conspecific (and congeneric) alarm odors, whereas learning to avoid dens with alarm odors from *Panulirus guttatus* likely increases fitness in reef-dwelling *Panulirus argus*, which leave their reef shelters to forage elsewhere during the night and then have to return to the reef to shelter during the day (Briones-Fourzán et al. 2008).

## Discussion

The present study basically describes differences and similarities in traits between *Panulirus* species that co-occur both regionally and locally. Although just showing that species differ phenotypically or ecologically is insufficient to assign those differences to the type of trade-off necessary to promote coexistence (Siepielski and McPeek 2010), differences in morphological, physiological, ecological, and behavioral traits can help generate hypotheses regarding niche differentiation and interspecific trade-offs that influence relative fitness differences and contribute to competitive inequalities (e.g. body size, fecundity, longevity, dispersal) that may lead to coexistence, especially between congeneric species that co-occur at local scales (Tilman 1994, Amarasekare 2003, HilleRisLambers et al. 2012).

*Panulirus argus* and *Panulirus guttatus* differ widely in their degree of habitat specialization and exhibit broad differences in many life history and ecological traits (e.g. larval and postlarval size and morphology, adult body size, fecundity, growth rate, movement range, behavior, susceptibility to predators) (Table 1). The large differences between *Panulirus argus* and *Panulirus guttatus* suggest the existence of important trade-offs leading to a stable coexistence of these two congeners. For example, although these congeners share the reef habitat, *Panulirus guttatus* is better at exploiting shelter and food resources in this habitat, but is more susceptible to predators relative to *Panulirus argus*. In contrast, *Panulirus inflatus* and *Panulirus gracilis* appear more similar in some traits (e.g. larval, postlarval, and adult size, diet) but they differ in other traits (e.g. fecundity, growth rate) and in habitat use, suggesting interspecific trade-offs that may contribute to competitive inequalities (Table 2). However, much remains to be investigated on the chemical ecology and behavioral traits of *Panulirus gracilis* and *Panulirus inflatus* that may influence their competitive abilities and susceptibility to predators (Table 2).

**Table 1.** Summary of differences in life-history and ecological traits between *Panulirus guttatus* and *Panulirus argus* living in sympatry in the Caribbean region.

Life history or ecological trait	*Panulirus guttatus*	*Panulirus argus*
Life-history style	Habitat specialist	Ontogenetic shifter
Larval and postlarval size	Larger	Smaller
Adult size	Smaller	Larger
Growth rate	Slower	Faster
Brood size	Smaller	Larger
Egg size	Larger	Smaller
Diet	Similar?	Similar?
Foraging habitats	Coral reef	Seagrass, rubble areas
Lifestyle	Highly sedentary	Highly mobile
Degree of gregariousness	Lower	Higher
Behavioral type	Shy	Bold
Susceptibility to predators	Higher	Lower
Response to conspecific alarm odors	Neutral	Avoidance
Response to congeneric alarm odors	Neutral	Avoidance
Competitive rank (in reef habitat)	Superior	Inferior

**Table 2.** Summary of differences in life history and ecological traits between *Panulirus inflatus* and *Panulirus gracilis* living in sympatry in the Eastern Central Pacific region.

Life-history or ecological trait	*Panulirus inflatus*	*Panulirus gracilis*
Life-history strategy	Habitat specialist?	Habitat generalist?
Larval and postlarval size	Similar	Similar
Adult size	Similar	Similar
Growth rate	Slower	Faster
Brood size	Smaller	Larger
Egg size	Larger	Smaller
Diet	Similar?	Similar?
Foraging habitats	Rocky areas	Rocky + gravel-sand areas
Lifestyle	Mobile	Highly mobile
Degree of gregariousness	?	?
Susceptibility to predators	?	?
Behavioral type	?	?
Response to conspecific alarm odors	?	?
Response to congeneric alarm odors	?	?
Competitive rank	Superior?	Inferior?

An intriguing hypothesis would be whether the substantial niche differentiation and apparent interspecific trade-offs between *Panulirus argus* and *Panulirus guttatus* relative to *Panulirus gracilis* and *Panulirus inflatus* reflect an earlier divergence of the former pair of species in the evolution of the genus. Several phylogenetic analyses (e.g. McWilliam 1995, Ptacek et al. 2001, Patek and Oakley 2003, George 2006) concur in that there are two major lineages in the radiation of *Panulirus*, with species in the first lineage representing an earlier radiation than species in the second lineage. The first lineage likely radiated from an ‘*argus*-like ancestor’ from which *Panulirus argus* split, possibly in the Mid-Miocene (18–8 mya) (Ptacek et al. 2001, George 2006). Morphological and molecular criteria place *Panulirus guttatus* in the first lineage as well, but the origin of this species remains uncertain as the late phyllosmata and the puerulus of *Panulirus guttatus* exhibit the long, spatulated antennae typical of species in the second lineage, probably as a result of early divergence (McWilliam 1995, Ptacek et al. 2001, George 2006). In contrast, *Panulirus gracilis* and *Panulirus inflatus* clearly belong to the second major lineage and these two species constitute a single clade, with *Panulirus inflatus* possibly splitting from *Panulirus gracilis* as recently as the late Miocene/Pliocene (8–2 mya) (George 2006).

However, differences between the two pairs of co-occurring species due to divergence times alone would imply that speciation occurred in ecological sympatry, and at least some speciation in the genus *Panulirus* appears to have been the result of vicarious events associated with major changes in oceanic currents (affecting larval dispersion) due to continental fig movements (George 2006). For example, a recent range expansion northward by *Panulirus gracilis* into the historical range of *Panulirus inflatus* could mean sympatry between these two species is much more recent than their time of divergence. Moreover, if life history traits such as growth rate, size at maturity, and fecundity tend to be more similar for species that occupy more similar microhabitats, this could partially explain the greater overlap between *Panulirus gracilis* and *Panulirus inflatus* than between *Panulirus argus* and *Panulirus guttatus*. For each pair of species, these hypotheses would have to be tested either via manipulative experiments involving removal or exclusion of one species to measure its impact on the other and vice versa, or by comparing locations where both species co-occur to locations where either species is absent.

More quantitative studies are also needed to determine how much overlap in the use of food resources truly exists between co-occurring species. Spiny lobsters are omnivorous consumers, but stomach content analyses suggest that some co-occurring species prefer different types of prey (e.g. Colinas-Sánchez and Briones-Fourzán 1990). The use of stable isotope analyses (SIA) (e.g. Waddington et al. 2008) may help to better define the trophic level of co-occurring *Panulirus* species. For example, a recent study using SIA in *Panulirus guttatus* and *Panulirus argus* from Puerto Morelos showed that small carnivores contribute more to the diet of adult *Panulirus guttatus* whereas small herbivores contribute more to the diet of reef-dwelling *Panulirus argus* (Segura-García et al. unpublished data). Similarly, high through-put DNA sequencing techniques (e.g. O’Rorke et al. 2012) may help identify a potential resource partitioning between the otherwise similar phyllosoma larvae of *Panulirus gracilis* and *Panulirus inflatus*.

Identifying mechanisms of coexistence for congeneric species that live in sympatry is an important issue for the establishment of marine protected areas by allowing identification of species that have broad or narrow habitat requirements (McPeek 1996, Acosta and Robertson 2003). It may also provide insight into how these species could respond to climate change and other human-mediated environmental impacts such as habitat loss, degradation, and fragmentation, as well as the introduction of invasive species (McPeek 1996, HilleRisLambers et al. 2012), all of which constitute pressing issues for the shallow-water *Panulirus* species (Briones-Fourzán and Lozano-Álvarez 2013).
